# HapX-Mediated Adaption to Iron Starvation Is Crucial for Virulence of *Aspergillus fumigatus*

**DOI:** 10.1371/journal.ppat.1001124

**Published:** 2010-09-30

**Authors:** Markus Schrettl, Nicola Beckmann, John Varga, Thorsten Heinekamp, Ilse D. Jacobsen, Christoph Jöchl, Tarek A. Moussa, Shaohua Wang, Fabio Gsaller, Michael Blatzer, Ernst R. Werner, William C. Niermann, Axel A. Brakhage, Hubertus Haas

**Affiliations:** 1 Division of Molecular Biology/Biocenter, Innsbruck Medical University, Innsbruck, Austria; 2 J. Craig Venter Institute, Rockville, Maryland, United States of America, and The George Washington University School of Medicine, Department of Biochemistry and Molecular Biology, Washington, D.C., United States of America; 3 Department of Molecular and Applied Microbiology, Leibniz Institute for Natural Product Research and Infection Biology (HKI), and Friedrich Schiller University Jena, Jena, Germany; 4 Department for Microbial Pathogenicity Mechanisms, Leibniz Institute for Natural Product Research and Infection Biology (HKI), and Friedrich Schiller University Jena, Jena, Germany; 5 Division of Biological Chemistry/Biocenter, Medical University Innsbruck, Innsbruck, Austria; University of Toronto, Canada

## Abstract

Iron is essential for a wide range of cellular processes. Here we show that the bZIP-type regulator HapX is indispensable for the transcriptional remodeling required for adaption to iron starvation in the opportunistic fungal pathogen *Aspergillus fumigatus*. HapX represses iron-dependent and mitochondrial-localized activities including respiration, TCA cycle, amino acid metabolism, iron-sulfur-cluster and heme biosynthesis. In agreement with the impact on mitochondrial metabolism, HapX-deficiency decreases resistance to tetracycline and increases mitochondrial DNA content. Pathways positively affected by HapX include production of the ribotoxin AspF1 and siderophores, which are known virulence determinants. Iron starvation causes a massive remodeling of the amino acid pool and HapX is essential for the coordination of the production of siderophores and their precursor ornithine. Consistent with HapX-function being limited to iron depleted conditions and *A. fumigatus* facing iron starvation in the host, HapX-deficiency causes significant attenuation of virulence in a murine model of aspergillosis. Taken together, this study demonstrates that HapX-dependent adaption to conditions of iron starvation is crucial for virulence of *A. fumigatus*.

## Author Summary

Due to its requirement for a wide range of cellular processes, iron is an essential nutrient for virtually every organism. The mammalian immune system utilizes iron-withholding mechanisms to deny access to free iron. Therefore, pathogens must overcome extreme iron limitation. Patients with suppressed immune systems due to cancer treatments, organ transplantation, or genetic disorders are at high risk of infection with the ubiquitously present fungal pathogen *Aspergillus fumigatus*. In this study we found that in *Aspergillus fumigatus* iron starvation results in drastic metabolic changes depending on the transcription factor HapX. During iron starvation, HapX functions include the repression of iron-consuming pathways to spare iron and activation of iron uptake by siderophores. Siderophores are small molecules able to “steal” iron from host molecules and have previously been shown to play a crucial role in the virulence of *Aspergillus fumigatus*. Genetic inactivation of HapX attenuates virulence of *Aspergillus fumigatus* in a murine model of aspergillosis, demonstrating that adaption to iron limitation is a crucial virulence determinant. The identification of numerous HapX-affected genes with a yet uncharacterized link to iron will aid in the further characterization of the metabolic pathways required for fungal adaption to iron starvation and virulence traits.

## Introduction

Iron is an essential nutrient for virtually every organism. The ability to exist in two redox states makes this metal an essential cofactor of proteins involved in numerous major cellular processes including respiration, amino acid metabolism and DNA metabolism. However, excess iron has the ability to generate toxic reactive species that can damage cellular components [1]. Despite its general abundance, the bioavailability of iron is very limited owing to its oxidation into insoluble ferric hydroxides by atmospheric oxygen. Consequently, all organisms have developed tightly regulated homeostatic mechanisms in order to balance uptake, storage and consumption of iron. Moreover, the mammalian immune system utilizes iron-withholding mechanisms to deny invading microorganism's access to free iron [2], [3]. Consequently, the control over access to iron is one of the central battlefields deciding the fate of an infection. Furthermore, iron starvation activates not only iron uptake but also virulence determinants in many prokaryotic and eukaryotic pathogens.

*Aspergillus fumigatus* is a typical ubiquitous saprophytic mold. Nevertheless, it causes life-threatening invasive disease especially in immuno-compromised patients and has become the most common airborne fungal pathogen of humans [4]. *A. fumigatus* lacks specific uptake systems for host iron sources as heme, ferritin, or transferrin [5]. However, it employs two high-affinity iron uptake systems, siderophore-assisted iron uptake and reductive iron assimilation, both of which are induced upon iron starvation. Siderophores are low molecular mass, ferric iron-specific chelators [6]. *A. fumigatus* excretes the siderophores fusarinine C (FsC) and triacetylfusarinine C (TAFC) to mobilize extracellular iron. Subsequent to chelation of iron, the ferric forms of FsC and TAFC are taken up by specific transporters [7]. For release of iron, the siderophores are intracellularly hydrolyzed [8] and the iron is transferred to the metabolic machinery or stored. *A. fumigatus* employs also intracellular siderophores: ferricrocin (FC) for hyphal storage and distribution of iron, and hydroxyferricrocin (HFC) for conidial iron storage [9], [10].

FsC is a cyclic tripeptide consisting of three *N^5^*-*cis*-anhydromevalonyl-*N^5^*-hydroxyornithine residues linked by ester bonds, TAFC is the *N^2^*-acetylated FsC, FC is a cyclic hexapeptide with the structure Gly-Ser-Gly-(*N^5^*-acetyl-*N^5^*-hydroxyornithine)_3_ and HFC is the hydroxylated FC [6]. The siderophore biosynthetic pathway is shown in Fig. S1. The first committed step in the biosynthesis of all four siderophores is hydroxylation of ornithine (Orn). Subsequently, the pathways for biosynthesis of TAFC and FC split involving acylation of *N^5^*-hydroxyornithine, assembly of siderophore-back bones by nonribosomal peptide synthetases (NRPS), and derivatization by acetylation or hydroxylation. Five *A. fumigatus* genes encoding respective enzyme activities have been identified [5], [10]: *sidA* (*N^5^*-ornithine-monooxygenase), *sidF* (*N^5^*-hydroxyornithine:*cis*-anhydromevalonyl coenzyme A-*N^5^*-transacylase), *sidC* (FC NRPS), *sidD* (fusarinine C NRPS) and *sidG* (fusarinine C:acetyl coenzyme A-*N^2^*-transacetylase). Elimination of both intra- and extracellular siderophores (*ΔsidA* mutants) results in absolute avirulence of *A. fumigatus* in a mouse model of pulmonary aspergillosis [5]. Deficiency in either extracellular (*ΔsidF* or *ΔsidD* mutants) or intracellular siderophores (*ΔsidC* mutants) causes partial attenuation of virulence [10]. Recently, siderophores have also been implicated in virulence of *Histoplasma capsulatum* and various phytopathogenic ascomycetes [11], [12], [13]. Consequently, the siderophore system represents an attractive target for antifungal therapy. However, not all fungi produce siderophores; notable examples are *Saccharomyces cerevisiae*, *Candida albicans* and *Cryptococcus neoformans* [6].

In agreement with iron playing an important role in the pathophysiology of *A. fumigatus*, increased bone marrow iron stores represent an independent risk factor for invasive aspergillosis [14]. Moreover, polymorphonuclear leukocytes inhibit growth of *A. fumigatus* conidia by lactoferrin-mediated iron depletion [15], and the human body produces proteins able to sequester fungal siderophores [16]. Consistently, the chelators EDTA and deferasirox enhance the efficacy of amphotericine B in animal models for invasive pulmonary aspergillosis [17], [18].

In *Aspergillus nidulans*, maintainance of iron homeostasis is mediated by two transcription factors, SreA and HapX, which are interconnected in a negative feed-back loop: SreA represses expression of *hapX* during iron sufficiency and HapX represses *sreA* during iron starvation [19], [20]. SreA is a DNA-binding GATA-factor whereas HapX functions by protein-protein interaction with the heterotrimeric CCAAT-binding factor. SreA represses iron uptake during iron sufficiency to avoid toxic effects and HapX represses iron-dependent pathways during iron starvation to spare iron. This regulatory circuit is largely conserved in *Schizosaccharomyces pombe* and orthologs to SreA and HapX are found in most fungal species; a notable exception is the fungal prototype *S. cerevisiae*, which employs entirely different regulators [6], [21].

We have previously demonstrated the role of SreA in repression of iron acquisition in *A. fumigatus* [22]. In this study we characterized the function of HapX and its interplay with SreA. We demonstrate that HapX function is crucial for the metabolic reprogramming required for adaption to iron starvation and for virulence of *A. fumigatus*.

## Results/Discussion

### HapX-deficiency decreases growth and sporulation specifically during iron starvation

**Figure 1:**
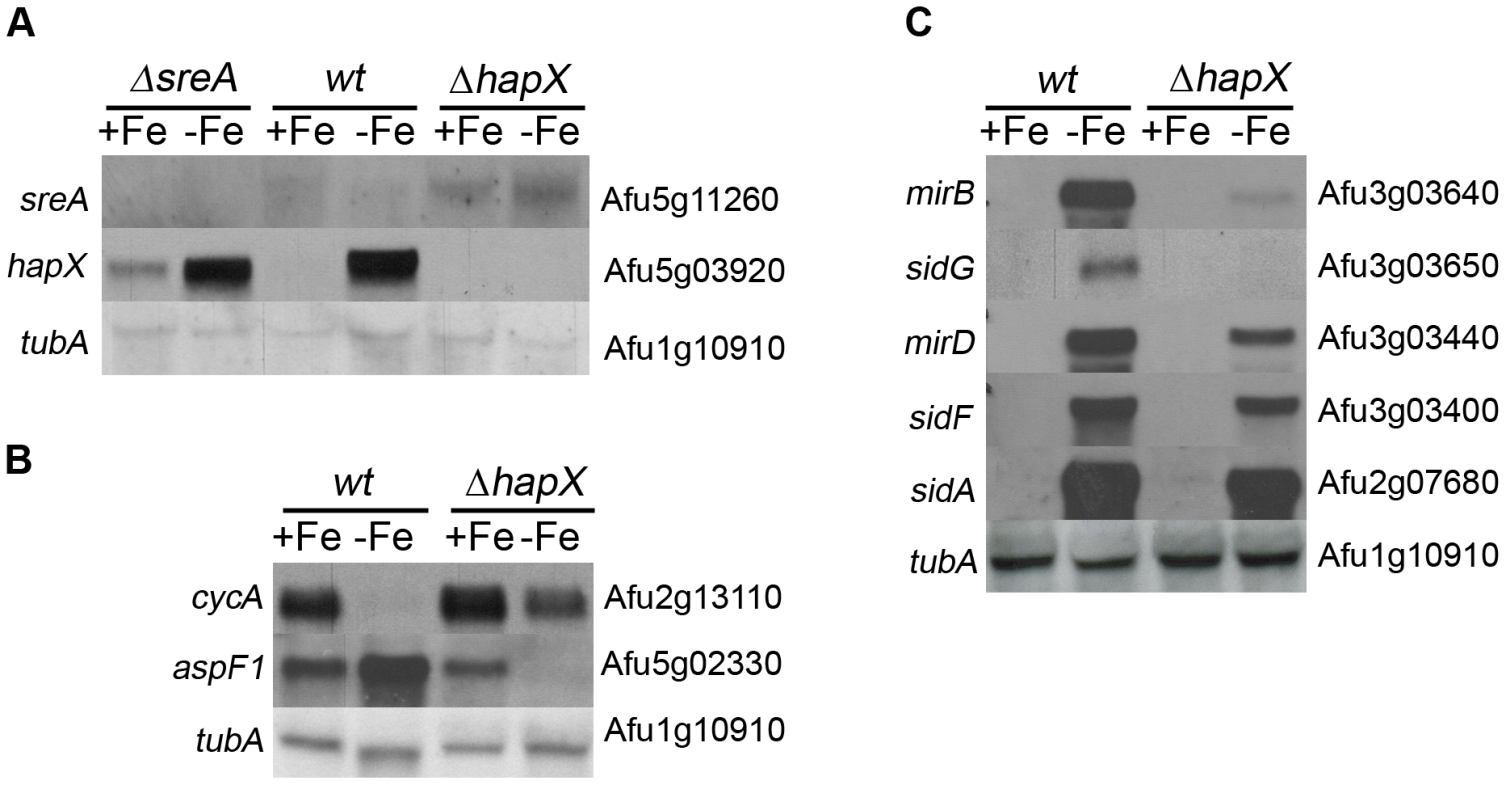
HapX affects iron regulation. (A) Mutual transcriptional control between HapX and SreA. (B) Examples for negative (*cycA*) and positive (*aspF1*) impact of HapX in iron regulation. (C) HapX is required for transcriptional activation of the siderophore system. *A. fumigatus wt*, ***Δ*** *sreA* and ***Δ*** *hapX* were grown in shake flask cultures under iron-replete (+Fe) and depleted (−Fe) conditions. Total RNA was isolated and subjected to Northern analysis of genes selected from the genome-wide expression profiling (Fig. S1 and S2). As a control for quality and quantity, RNAs were hybridized with the ß-tubulin encoding *tubA* gene.

**Figure 2:**
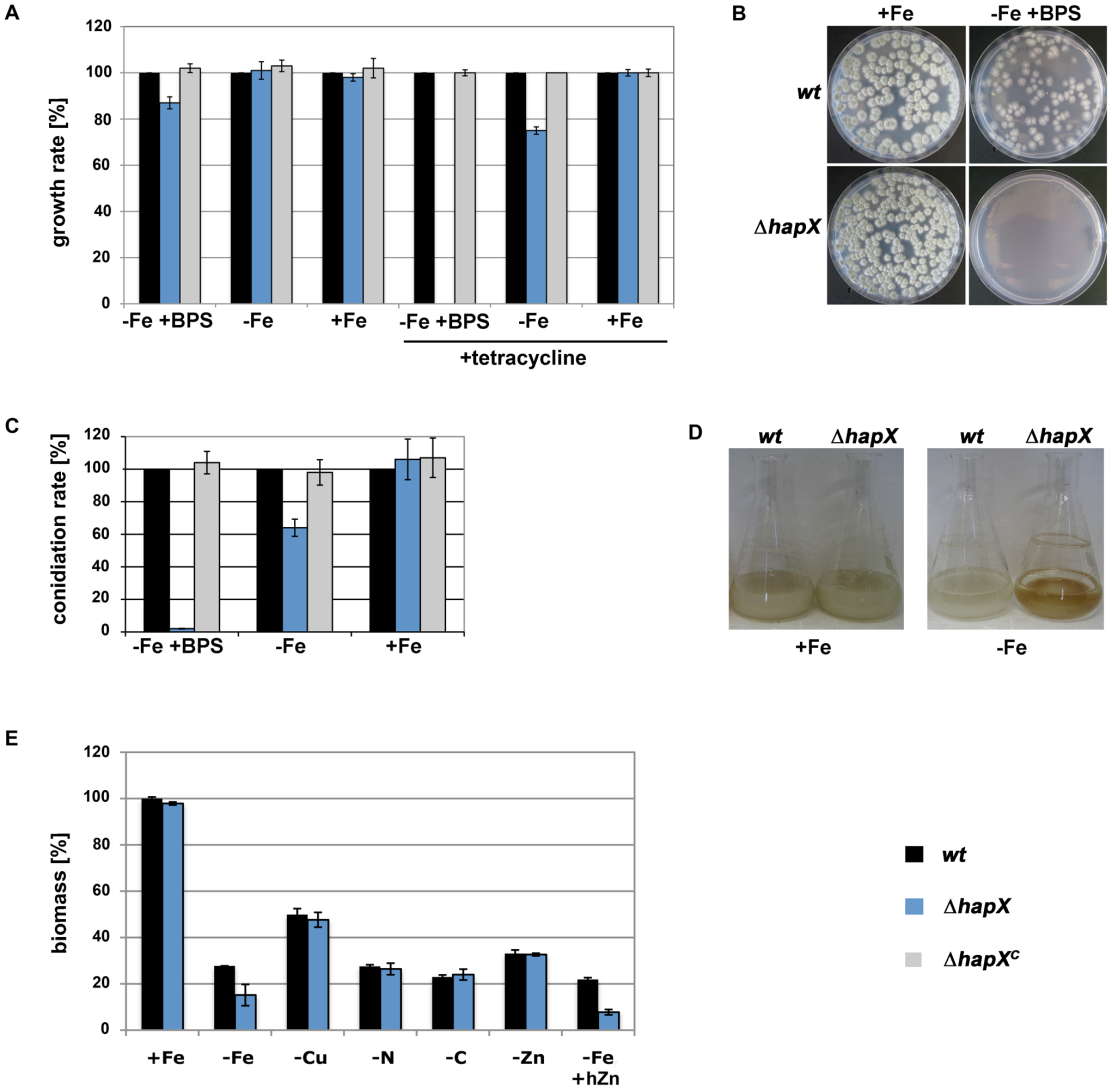
During iron depleted but not iron-replete conditions, HapX-deficiency impairs growth in liquid and solid media, colony formation from single conidia, conidiation, resistance to zinc and tetracycline, and causes reddish pigmentation. (A) Growth on solid media with and without tetracycline: conidia were point-inoculated on plates reflecting harsh iron starvation (−Fe +BPS), iron starvation (−Fe) and iron-replete conditions (+Fe). Radial growth was recorded after 48h of growth at 37°C and normalized to that of *wt* grown under the same condition. (B) Colony formation from single conidia: approximately 100 conidia were plated and photographs were taken after growth for 48h at 37°C. (C) Conidiation: conidia production by 1 cm^2^ of mycelia after growth for 120h of at 37°C was recorded and normalized to that of *wt* under the same condition. (D) Hyphal pigmentation: photographs of liquid cultures were taken after 24h of growth at 37°C. (E) Growth in liquid media with and without limitations or zinc excess: biomass production of 10^8^ conidia inoculated in 100 ml media was scored after 24 h of growth at 37°C and normalized to that of *wt* in +Fe. For limitation of carbon (−C), nitrogen (−N), zinc (−Zn) and copper (−Cu), the growth medium contained 0.1% glucose, 2 mM glutamine, 1 mM ZnSO_4_ and no added copper, respectively, which decreased the biomass production of the *wt* to about the same extent as iron limitation. High zinc-medium (hZn) contained 0.5mM ZnSO_4_. The data in (A), (C), and (E) represent the means ± standard deviations from three independent experiments.

In *A. nidulans*, HapX has been shown to repress iron-dependent pathways during iron starvation [20]. The *A. fumigatus* HapX ortholog displays 70% overall identity and contains all typical features common to this class of transcription factors: an N-terminal 17 amino acid motif, which is essential for interaction with the CCAAT-binding complex, a bZip domain, and three cysteine-rich regions, which are potentially involved in iron-sensing. Genome-wide transcriptional profiling revealed that the transcript level of the *A. fumigatus hapX* ortholog (Afu5g03920) is SreA-dependently down-regulated in a shift from iron depleted to iron-replete conditions [22]. In agreement, Northern analysis demonstrated up-regulation of the *hapX* transcript level under steady-state iron depleted compared to iron-replete conditions and partial derepression during iron-replete conditions in a *ΔsreA* mutant (Fig. 1A). This expression pattern matches that of the *A. nidulans* ortholog [20]. In order to analyze the function of HapX in *A. fumigatus*, a deletion mutant (*ΔhapX*) was generated as described in *Methods*. Consistent with undetectable expression of *hapX* during iron sufficiency in *wt* (Fig. 1A), *ΔhapX* displayed no significant difference to the *wt* with respect to conidiation and growth rate on solid or liquid media during iron sufficiency (Fig. 2). In contrast, *ΔhapX* showed mildly reduced radial growth on solid media (Fig. 2A) and was not able to form colonies from single conidia in the presence of the iron chelator bathophenanthroline disulfonate (BPS) (Fig. 2B). Furthermore, *hapX* deletion decreased conidiation to 62% of the *wt* during iron starvation and 4% during iron starvation in the presence of BPS (Fig. 2C). In iron-starved liquid culture, *hapX* deletion decreased the biomass production to 58% of the *wt* (Fig. 2E) and caused a reddish pigmentation of the mycelia (Fig. 2D). Reintegration of a functional *hapX* copy at the *hapX* locus in the *ΔhapX* strain, yielding strain *ΔhapX^C^*, cured these and all other defects (Fig 2 and data not shown), which demonstrates that the *ΔhapX* phenotype is a direct result of the loss of HapX activity. Notably, germination of *ΔhapX* was *wt*-like under iron-replete and depleted conditions (data not shown) demonstrating that the phenotypes of *ΔhapX* are caused by growth defects. Limitation of nitrogen, carbon, copper, or zinc decreased biomass production of *ΔhapX* and *wt* to similar extents (Fig. 2E), which indicates that inactivation of HapX does not result in general sensitivity to starvation but in particular to iron starvation.

### HapX and SreA are interconnected by a negative regulatory feedback loop

In line with the growth defect of ***Δ*** *hapX* under iron starvation but not iron sufficiency, expression of *hapX* was repressed by iron at the transcriptional level, partly dependent on SreA (Fig. 1A). In turn, HapX repressed *sreA* during iron starvation (Fig. 1A). A similar expression pattern was described previously for the *hapX* and *sreA* orthologs of *A. nidulans* and *S. pombe* [20], [21].

### HapX is required for repression of genes during iron starvation

Genome-wide transcriptional profiling revealed that expression of *hapX* is repressed within ≤30 minutes in a shift from iron depleted to iron-replete conditions [22], which predicts that HapX targets also respond quickly to the availability of iron. This HapX feature allowed analysis of short-term effects of *hapX* deletion. In order to identify the genes that are negatively affected by HapX at the transcript level, we therefore searched by genome-wide transcriptional profiling for genes fulfilling three criteria: (i) up-regulation in a 1h-shift from iron starvation to iron sufficiency in *wt* (identification of genes repressed by iron starvation), (ii) decreased up-regulation in a 1h-shift from iron starvation to iron sufficiency in ***Δ*** *hapX* compared to *wt* (identification of genes showing a short-term response to HapX inactivation), and (iii) up-regulation during steady-state iron starved growth in ***Δ*** *hapX* compared to *wt* (identification of genes showing a long-term response to HapX inactivation). This strategy is supposed to select for rather direct effects of HapX inactivation.

Among the 131 genes negatively affected by HapX (Table S1 in Supporting Information S1 and Table 1A), 34% can be directly assigned to iron-dependent pathways including respiration, TCA cycle, amino acid metabolism, iron-sulfur-cluster biosynthesis, heme biosynthesis, oxidative stress detoxification, biotin synthesis (Afu6g03670), vacuolar iron storage (CccA, Afu4g12530), and iron regulation (SreA, Afu5g11260). This gene set included the orthologs of all five previously identified *A. nidulans* HapX-repressed genes [20]: *cycA* (cytochrome C, respiration, Afu2g13110), acoA (aconitase, TCA cycle, Afu6g12930), *hemA* (α-amino-levulinic acid synthase; heme biosynthesis, Afu4g11400), *lysF* (homoaconitase, lysine biosynthesis, Afu5g08890), and *sreA* (repressor of iron uptake). A representative Northern analysis of *cycA* is displayed in Fig. 1B.

**Table 1:** Categorization of the genes affected by HapX.

A				
Process	Genes	Mitochondrial	Transmembrane	> in *ΔsreA*
**iron-dependent**	44	35 (79%)	2 (5%)	29 (86%)
respiration	20	20 (100%)	0	10 (70%)
aa metabolism	4	3 (75%)	0	4 (100%)
FeS-cluster biogenesis	2	2 (100%)	0	2 (100%)
heme metabolism	3	2 (67%)	1 (33%)	3 (100%)
TCA cycle	6	6 (100%)	0	6 (100%)
cellular detoxification	3	1 (33%)	0	2 (67%)
other processes	6	1 (17%)	1 (17%)	2 (33%)
**ribosomal biogenesis**	30	0	0	2 (7%)
**aa metabolism**	7	2 (29%)	0	2 (29%)
**additional mitochondrial**	4	4 (100%)	1 (25%)	1 (25%)
**additional regulatory**	12	0	0	5 (42%)
**additional non-regulatory**	17	0	3 (29%)	5 (29%)
**unknown function**	17	0	0	5 (29%)
**Total**	**131**	**41 (31%)**	**6 (5%)**	**50 (38%)**

B				
Process	Genes	Mitochondrial	Transmembrane	SreA targets
**siderophore metabolism**	8	0	3 (38%)	8 (100%)
**aa metabolism**	9	0	0	0
**protein degradation/uptake**	8	0	5 (63%)	4 (50%)
**acetyltransferases**	5	0	0	4 (80%)
**carbohydrate metabolism**	14	0	4 (295)	5 (36%)
**lipid/ergosterol/fatty acid**	6	0	0	2 (33%)
**GPI**	3	0	0	1 (33%)
**regulatory/signalling**	11	0	0	1 (10%)
**transmembrane proteins**	8	0	8 (100%)	3 (38%)
**other functions**	36	0	0	1
**hypothetical proteins**	31	0	0	10 (32%)
**Total**	**139**	**0**	**20 (14%)**	**38 (27%)**

(A) Genes repressed during iron starvation in *wt* and derepressed in ***Δ*** *hapX* (from Fig. S1), and (B) genes induced by iron starvation in *wt* and down-regulated in ***Δ*** *hapX* (from Fig. S2).

The majority of the cellular iron-consuming pathways, e.g. heme biosynthesis, iron-sulfur cluster biosynthesis, respiration, TCA cycle, is localized in mitochondria, which might explain the co-regulation of mitochondrial components that are not directly iron-dependent, e.g. the mitochondrial processing peptidase (Afu1g14200), which is essential for import of all mitochondrial matrix proteins. Strikingly, 31% (n = 41) of the genes negatively affected by HapX encode proteins that are localized in mitochondria (Table S1 in Supporting Information S1 and Table 1A), which indicates a significant impact of HapX on mitochondrial metabolism.

23% (n = 30) of the identified genes repressed during iron starvation in a HapX-dependent manner are involved in ribosomal biogenesis and translation (Table S1 in Supporting Information S1 and Table 1A). These data might reflect the iron-dependence of the translation machinery due to the essentiality of iron-sulfur clusters for function of Rli1 (RNase L inhibitor, Afu1g10310). Because of its fundamental role in translation initiation and ribosome biogenesis, RLI1 is one of the most conserved proteins present in all organisms except eubacteria and it is essential in all organisms tested [23]. Consistent with its iron-dependence, Rli1 expression is repressed during iron starvation in a HapX-dependent manner (Table S1 in Supporting Information S1). The down-regulation of translation during iron starvation indicates a slow-down of the entire metabolism, which might serve extended cellular survival.

Among the 131 *A. fumigatus* genes negatively affected by HapX, 21 have orthologs in *S. pombe* (Table S1 in Supporting Information S1), which are negatively affected by the HapX ortholog Php4 [24]. *S. cerevisiae* lacks an HapX ortholog and down-regulation of iron-dependent pathways during iron starvation is mediated by the paralogous proteins Cth1 and Cth2, which promote decay of target mRNA's during iron starvation [25]. A total of 21 of HapX-repressed genes have orthologs in *S. cerevisiae*, which are repressed during iron starvation *via* Cth1/2 (Table S1 in Supporting Information S1). Taken together, all three fungal species repress 15 orthologous genes during iron starvation (Table S1 in Supporting Information S1). All 15 deduced gene products are involved in iron-dependent pathways including respiration, iron sulfur cluster biosynthesis, TCA cycle, amino acid metabolism and translation and all are localized in mitochondria with exception of Rli1 and the leucine biosynthetic enzyme Leu1 (Table S1 in Supporting Information S1). These data underscore the evolutionary conservation of iron-sparing in different fungal species.

Comparison of the genes negatively affected by HapX with the previously identified SreA regulon [22] displayed no overlap (Table S1 in Supporting Information S1). However, 38% of these genes were previously found to be up-regulated indirectly by SreA-deficiency; i.e., in a shift from iron starvation to iron sufficiency these genes were up-regulated in ***Δ*** *sreA* only at late time points. As iron represses expression of *hapX* at the transcriptional level (see above) and most likely also post-translationally, as shown for its orthologs in *A. nidulans* and *S. pombe* [20], [26], these data suggest that the up-regulation of these genes in ***Δ*** *sreA* cells is caused by inactivation of HapX through the iron overload in ***Δ*** *sreA*.

### HapX is involved in induction of genes during iron starvation

To identify the genes affected positively by HapX, the inverse criteria compared to the screening for HapX-repressed genes by transcriptional profiling were applied (see above): (i) down-regulation in a shift from iron starvation to iron sufficiency in *wt*, (ii) decreased down-regulation in a shift from iron starvation to iron sufficiency in ***Δ*** *hapX* compared to *wt*, and (iii) down-regulation during steady-state iron starved growth in ***Δ*** *hapX* compared to *wt*.

Genes affected positively by HapX or its ortholog have been described for neither *A. nidulans* nor *S. pombe* yet. However, the transcriptional profiling identified 139 such genes in *A. fumigatus*, which are mainly involved in siderophore metabolism, amino acid metabolism, protein degradation and uptake, carbohydrate metabolism, and lipid metabolism (Table S2 in Supporting Information S1 and Table 1B). Strikingly, 27% of these genes were previously found to be SreA targets, i.e. repressed during iron sufficiency by SreA [22], e.g., genes involved in siderophore metabolism (Table S2 in Supporting Information S1 and Table 1B). As *hapX* deletion derepressed expression of *sreA* during iron starvation (see above), *hapX* deletion might repress SreA-targets indirectly *via* its transcriptional derepression of *sreA*.

However, *hapX* deletion affected expression of various SreA-target genes differently (Table S2 in Supporting Information S1, Fig. 1C). HapX-deficiency drastically reduced the transcript levels of the putative siderophore transporter-encoding *mirB* and the siderophore-biosynthetic *sidG* but had only minor effects on the siderophore transporter-encoding *mirD* and siderophore-biosynthetic *sidA* and *sidF*, which indicates SreA-independent effects. In line, 73% of the genes negatively affected by *hapX* deletion do not appear to be SreA targets (Table S2 in Supporting Information S1 and Table 1B). A prominent example is one of the major allergens of *A. fumigatus*, the ribotoxin Aspf1 (Afu5g02330) [27]. The microarray data (Table S2 in Supporting Information S1) and Northern analysis revealed that the transcriptional up-regulation of AspF1 during iron starvation is strictly dependent on HapX (Fig. 1B) and not affected by SreA as shown previously [22]. AspF1 is cytotoxic and was shown to induce apoptosis of human immature dendritic cells, which indicates that it is involved in immune evasion of *A. fumigatus* [28]. However, AspF1 was previously shown to be dispensable for virulence of *A. fumigatus* in a murine model of aspergillosis [29]. A possible explanation for this discrepancy is that the immunosuppressive regimen used in the murine model interferes with the ability of the immune system to preferentially identify the mutant strains. As AspF1-activity is neither iron-dependent nor directly involved in iron acquisition, iron starvation might serve in this case as a signal for expression of a general virulence determinant not related to iron uptake. On the other hand, AspF1 might indirectly increase iron supply of *A. fumigatus* during the interaction with predators and hosts via cellular iron release due its cytotoxicity.

To further investigate the link between HapX and SreA activities we aimed to generate an *A. fumigatus* mutant lacking both regulators. However, several approaches to generate a ***Δ*** *sreA* ***Δ*** *hapX* double mutant failed indicating that deletion of *sreA* and *hapX* is synthetically lethal as shown previously in *A. nidulans* (Hortschansky et al., 2007), which underlines the importance of iron regulation.

### Genomic organization of the genes affected by HapX

Genes involved in common pathways tend to be genomically clustered in filamentous fungi. Therefore it is interesting to note that among the genes affected by HapX, 41 are organized in gene clusters (Tables S3 and S4 in Supporting Information S1). Interestingly, the AspF1-encoding gene is neighboured by a co-regulated gene encoding a hypothetical protein (Afu5g02320) and this gene organization is conserved in various fungal species, e.g., *Neosartorya fischeri*, *Aspergillus clavatus*, *Microsporum canis*, and *Arthroderma benhamiae* (data not shown). The acetyl transferase-encoding gene Afu5g00720, one of the clustered genes, was subjected to deletion analysis. Due to its expression pattern it appeared to be a good candidate for the still unidentified acetyl transferase required for FC biosynthesis (Fig. S1). However, the deletion did not reveal any phenotype (data not shown).

### HapX inactivation decreases production of TAFC and FC

**Figure 3:**
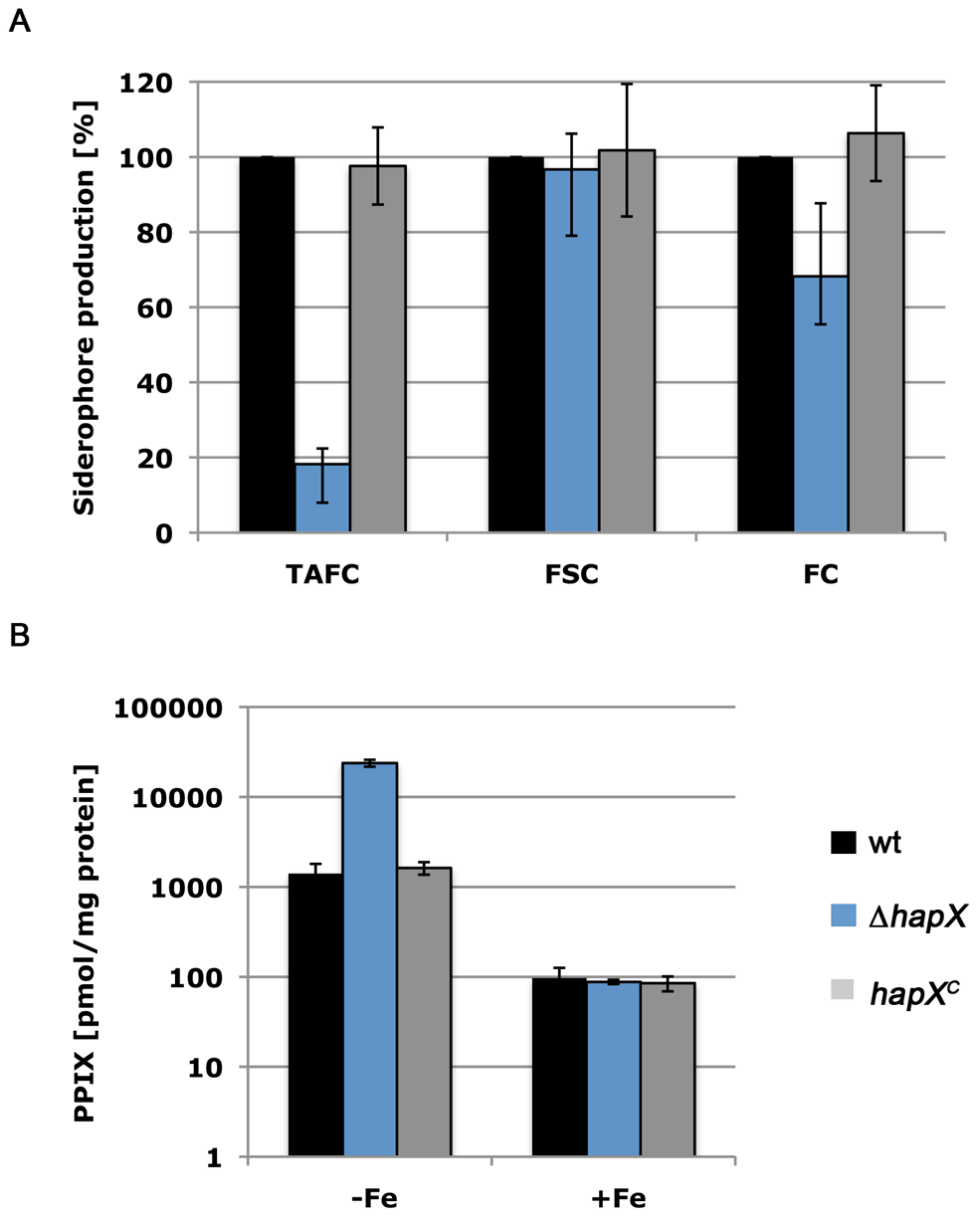
HapX-deficiency decreases production of TAFC and FC but increases cellular accumulation of PpIX. (A) Quantification of siderophore production after growth for 24 hours at 37°C under −Fe conditions normalized to that of *wt*. (B) Quantification of the PpIX content after growth for 24h at 37°C under iron-replete (+Fe) and depleted (−Fe) conditions. The data represent the mean ± standard deviation of three individually performed experiments.

*A. fumigatus* excretes the siderophores FsC and TAFC in roughly equal amounts (Fig S2). Inactivation of HapX did not substantially alter FsC production but reduced TAFC production to 18% of the *wt* (Fig. 3A). TAFC is derived from FsC by SidG-catalyzed *N^2^*-acetylation [22]. (Fig. S1). Consistent with the reduction of TAFC production, the *sidG* (Afu3g03650) transcript level was drastically reduced in ***Δ*** *hapX* as shown by Northern and microarray analyses (Fig. 1, Table S2 in Supporting Information S1). Blocking TAFC synthesis by inactivation of SidG has previously been shown to result in increased FsC production [22]. As FsC production was not increased in ***Δ*** *hapX*, it appears unlikely that SidG is the only siderophore biosynthetic activity affected in ***Δ*** *hapX*. In agreement, the microarray analyses (Table S2 in Supporting Information S1 and Table 2) revealed transcriptional down-regulation of other FsC biosynthetic enzymes such as SidF (Afu3g03400) and SidI (Afu1g17190). Moreover, supply of the siderophore precursor Orn might play a role in siderophore production (see below).

**Table 2:** Iron starvation remodels the free amino acid pool composition in *A. fumigatus*.

aa	*wt*			*ΔhapX/wt*		*ΔsreA/wt*		*ΔsidA/wt*	
	+Fe	−Fe	−/+Fe	+Fe	−Fe	+Fe	−Fe	+Fe	−Fe
**Ala**	34.15±0.93	8.63±1.29	***0.25***	1.07	**0.41**	0.98	1.12	0.92	0.78
**Arg**	1.28±0.11	13.20±1.75	***10.31***	1.24	**1.59**	0.84	1.13	1.22	1.13
**Asn**	0.82±0.04	2.46±0.29	***3.01***	1.05	1.43	1.30	1.03	0.89	0.84
**Asp**	3.61±0.40	3.47±0.33	0.96	1.14	1.16	1.30	1.05	0.90	**0.65**
**Gln**	7.03±0.25	37.01±2.75	***5.26***	0.90	**0.58**	0.73	0.91	1.05	0.84
**Glu**	42.14±1.46	18.29±0.54	**0.43**	0.87	0.93	0.98	1.02	1.05	0.79
**Gly**	1.53±0.10	1.03±0.02	0.67	1.18	***7.41***	0.99	1.10	1.01	**0.50**
**His**	0.18±0.06	1.69±0.10	***9.17***	**1.78**	**1.96**	1.33	1.01	1.11	0.85
**Ile**	0.34±0.01	0.33±0.07	1.00	1.26	1.21	1.21	1.03	0.85	**0.64**
**Leu**	0.38±0.05	0.50±0.06	1.33	1.39	**1.60**	1.32	0.90	1.03	**0.64**
**Lys**	1.55±0.20	3.45±0.32	**2.23**	1.2	***3.14***	0.96	1.04	1.01	0.70
**Met**	0.06±0.01	0.09±0.02	**1.68**	1.17	0.78	1.00	1.11	1.00	0.67
**Orn**	0.76±0.01	5.24±0.49	***6.91***	**1.88**	***0.08***	**0.38**	0.98	**1.87**	***3.83***
**Phe**	0.11±0.03	0.18±0.03	**1.63**	1.36	**1.67**	1.18	0.89	1.00	0.83
**Ser**	1.92±0.04	1.65±0.09	0.86	1.10	1.42	1.10	0.98	1.09	0.71
**Thr**	1.44±0.06	1.51±0.09	1.05	1.23	1.19	1.46	1.09	0.93	0.81
**Trp**	0.03±0.02	0.04±0.02	**1.55**	1.33	**2.75**	1.00	1.00	0.67	1.00
**Tyr**	0.25±0.07	0.37±0.04	1.47	1.36	1.43	1.36	0.95	1.00	1.22
**Val**	2.44±0.03	0.85±0.22	**0.35**	1.25	1.27	**1.57**	0.94	0.90	**0.56**

Individual amino acid pools are given in % of the total free amino acids. Amino acid pools up-regulated >1.5- and >3-fold in mutant strain versus *wt* are in **bold** and ***bold***, respectively; amino acid pools down-regulated >1.5- and >3-fold is marked in **bold** and ***bold***, respectively.

The transcriptional profiling (Table S2 in Supporting Information S1) also revealed down-regulation in ***Δ*** *hapX* of the NRPS SidC (Afu1g17200), which is essential for FC biosynthesis. Consistently, the FC content of ***Δ*** *hapX* was decreased to 68% of the *wt*.

In *A. nidulans*, HapX inactivation also decreased TAFC production but increased FC production [20]. Despite the general similarity of iron homeostasis-maintaining mechanisms of these two *Aspergillus* species, these data reveal differences.

### HapX inactivation results in excessive cellular accumulation of the iron-free heme precursor protoporphyrin IX (PpIX)

In contrast to *wt*, ***Δ*** *hapX* mycelia displayed a reddish pigmentation concomitant with red autofluorescence during iron depleted but not iron-replete conditions (Fig. 2D and data not shown), which is characteristic for accumulation of PpIX, the iron free precursor of heme [20]. Accordingly, the PpIX content of ***Δ*** *hapX* resembled the *wt* during iron-replete conditions but was 17-fold increased during iron starvation (Fig. 3B). These data indicate derepression of heme biosynthesis during iron starvation in ***Δ*** *hapX*, consistent with the expression profile of genes encoding 5-aminolevulinate synthase (Afu5g07750), ferrochelatase (Afu5g07750) and a putative heme transporter (Afu4g11400) revealed by the microarray analysis Table S1 in Supporting Information S1 and Table 1A).

### HapX is involved in remodeling of the cellular free amino acid pool in response to iron-starvation

The transcription profiling indicated changes in iron-dependent and -independent steps of the amino acid metabolism in response to HapX inactivation (Tables S1 and S2 in Supporting Information S1, Table 1). To gain further insight, we measured the relative composition of the free amino acid pool in *wt*, ***Δ*** *hapX*, ***Δ*** *sreA*, and ***Δ*** *sidA* during iron sufficiency and starvation (Table 2 and Table S5 in Supporting Information S1). In *wt*, iron starvation caused a dramatic remodeling of the composition of free amino acid pool: the relative amounts of nine amino acids (Arg, Asn, Gln, His, Lys, Met, Orn, Phe, and Trp) increased whereas that of three amino acids (Ala, Glu and Val) decreased >1.5-fold. ***Δ*** *hapX* and ***Δ*** *sidA* displayed differences compared to *wt* mainly during iron starvation, whereas ***Δ*** *sreA* showed differences mainly during iron sufficiency. This is in line with the expression pattern of the deleted genes: *hapX* and *sidA* are repressed while *sreA* is induced by iron (Fig. 1).

**Figure 4:**
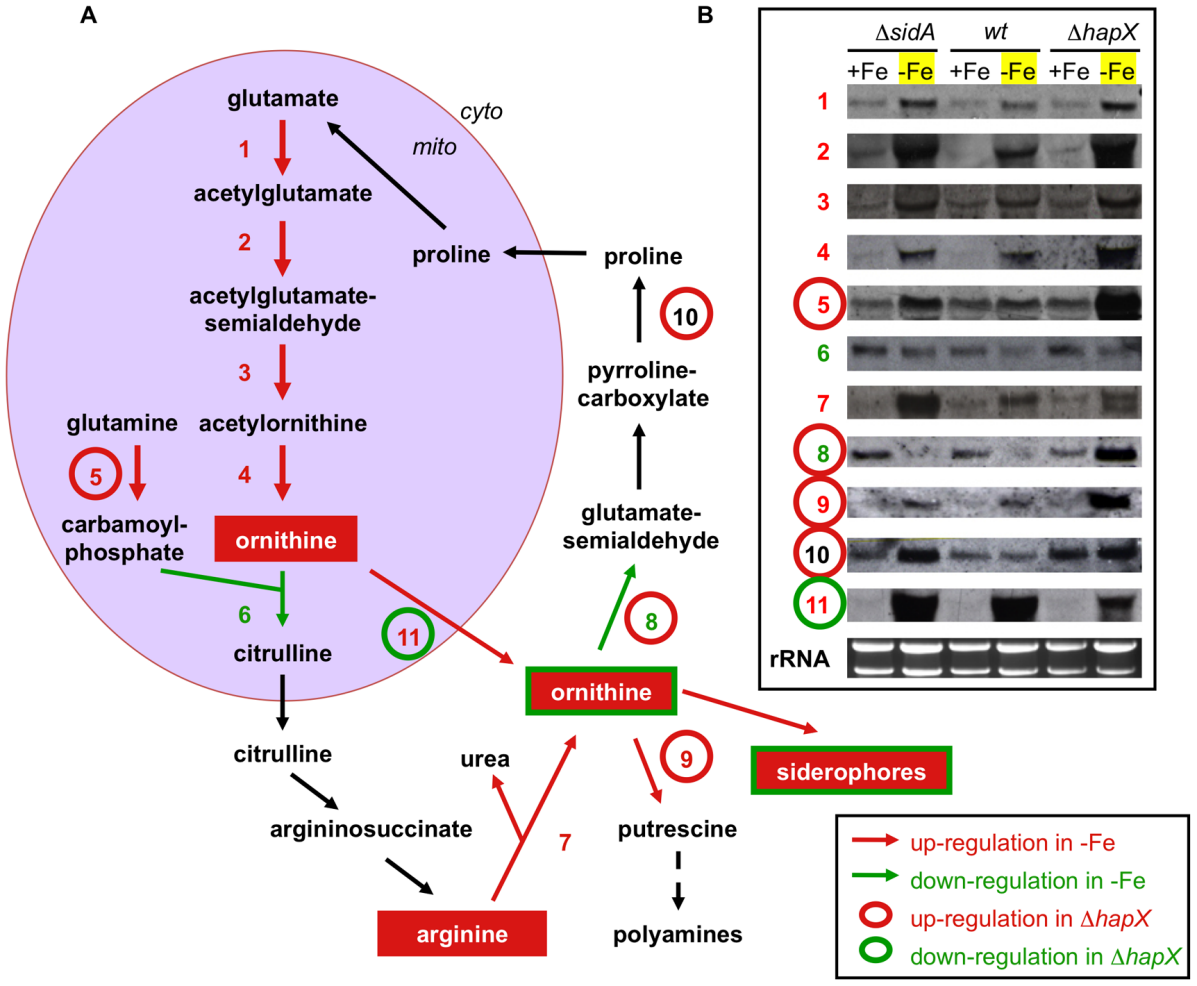
Iron starvation transcriptionally up-regulates biosynthesis of ornithine in a HapX-dependent manner. (A) Schematic representation of ornithine/arginine metabolism in *A. fumigatus*. Ornithine biosynthesis takes place in mitochondria (mito). Ornithine and citrulline are shuttled to the cytoplasm (cyto) and serve as precursors for arginine, siderophores and polyamines. Enzymatic steps within the pathways are numbered and corresponding to the Northern analysis in (B): 1, acetylglutamate synthetase (Afu2g11490) 2, acetylglutamate kinase and glutamate-5-semialdehyde dehydrogenase (Afu6g02910); 3, acetylornithine aminotransferase (Afu2g12470); 4, arginine biosynthesis bifunctional enzyme (Afu5g08120); 5, carbamoylphosphate synthase(Afu5g06780); 6, ornithine carbamoyltransferase (Afu4g07190); 7, arginase (Afu3g11430); 8, ornithine aminotransferase (Afu4g09140); 9, ornithine decarboxylase (Afu4g08010); 10, pyrroline carboxylate dehydrogenase (Afu6g08750); 11, ornithine transporter (Afu8g02760). Red and green arrows mark enzymatic steps transcriptionally up and down-regulated, respectively, by iron starvation in the *wt* as shown in (B); Red and green circles mark genes, which are transcriptionally up- and down-regulated, respectively, in a *ΔhapX* strain as shown in (B). (B) For Northern analysis, *wt*, *ΔsidA* and Δ*hapX* strains were grown for 24h at 37°C in under iron-replete (+Fe) and depleted (−Fe) conditions, respectively.

During iron starvation, siderophore production reaches up to 10% of the biomass and the major amino acid precursor for siderophore biosynthesis is Orn. The 6.9-fold increase of the Orn pool during iron starvation compared to iron sufficiency in *wt* indicates that the enormous Orn demand for siderophore biosynthesis is matched by active up-regulation of Orn biosynthesis during iron starvation and not by de-repression of Orn biosynthesis *via* its consumption, which could be expected to decrease the Orn pool. Consistently, blocking Orn consumption for siderophore biosynthesis by inactivation of the Orn hydroxylase SidA (***Δ*** *sidA*) caused a further 2.9-fold increase of the Orn pool during iron starvation compared to *wt*. Orn is synthesized from glutamate or from Orn-derived Arg (Fig. 4). Consistent with the amino acid analysis, Northern analysis confirmed transcriptional up-regulation of several key enzymes of the Orn/Arg biosynthetic pathway not only in *wt* but also in ***Δ*** *sidA*, which does not consume Orn for siderophore biosynthesis (Fig. 4). Strikingly, the Orn pool was 12.5-fold decreased in ***Δ*** *hapX*, while Arg was 2.0-fold increased (Table 2). Consequently, the Arg∶Orn ratio changed from 1.5 in *wt* to 49.9 in ***Δ*** *hapX*. Northern analysis demonstrated *wt*-like transcriptional up-regulation of most key enzymes of the Orn/Arg pathway in ***Δ*** *hapX* (Fig. 4). In perfect agreement with the microarray data (Tables S1 and S2 in Supporting Information S1), however, transcript levels of four involved enzymes were changed in ***Δ*** *hapX* during iron starvation (Fig. 4). Consistent with the increased Arg∶Orn ratio in ***Δ*** *hapX*, transcriptional up-regulation of the carbamoyl-phosphate-synthetase (Afu5g06780) and transcriptional down-regulation of the mitochondrial ornithine exporter AmcA (Afu8g02760) in ***Δ*** *hapX* during iron starvation is expected to promote production of Arg relative to Orn; up-regulation of ornithine aminotransferase: (Afu4g09140), ornithine decarboxylase (Afu4g08010), and proline oxidase (Afu6g98760) indicates increased consumption of ornithine for purposes other than biosynthesis of siderophores. Taken together, these data indicate that HapX is required for the up-regulation of the Orn pool to fuel siderophore biosynthesis. Therefore, the largely decreased Orn pool of ***Δ*** *hapX* might be in part responsible for the reduced production of TAFC and FC in addition to the transcriptional down-regulation of siderophore biosynthetic enzymes (see above). Consistently, derepression of siderophore biosynthesis during iron sufficiency by deletion of *sreA* (***Δ*** *sreA*), when HapX is inactive, decreased the Orn pool to 38% of *wt* (Table 2).

The 4.9-fold increased lysine pool in ***Δ*** *hapX* compared to *wt* during iron starvation is consistent with transcriptional up-regulation of the lysine biosynthetic enzymes homoaconitase LysF (Afu5g08890) and homocitrate synthase (Afu4g10460) (Table S1 in Supporting Information S1). The iron-dependence of LysF might explain the 0.7-fold decrease of the lysine pool in ***Δ*** *sidA* (Table 2) because lack of siderophore biosynthesis in ***Δ*** *sidA* causes increased iron starvation, which in turn down-regulates and inactivates iron-dependent pathways.

In the first commited step of heme biosynthesis, 5-aminolevulinate is synthesized from glycine and succinyl-CoA by HemA. As HemA expression and the heme biosynthetic pathway is derepressed during iron starvation in ***Δ*** *hapX* (see above), the 7.5-fold increase in the glycine pool might indicate synchronization of heme biosynthesis and supply of its precursor glycine by HapX (Table 2). Here, HapX would formally function as a repressor, whereas it acts as an activator for biosynthesis of siderophores and their precursor ornithine. We have previously shown that iron starvation down-regulates heme biosynthesis [22]. Therefore, the possibility of a regulatory link of glycine and heme biosynthesis is underlined by the 0.7-fold decrease of the glycine pool during iron starvation compared to iron sufficiency in *wt* and the further 0.5-fold decrease in ***Δ*** *sidA*.

Recently, iron starvation was found to influence the composition of the free amino acid pool in *S. cerevisiae* only mildly [30] with low concordance to *A. fumigatus* (Table S6 in Supporting Information S1). The difference might be due to the different life styles of *A. fumigatus* and *S. cerevisiae* and of course the inability of *S. cerevisiae* to synthesize siderophores.

### HapX inactivation increases the mitochondrial DNA (mtDNA) content and decreases resistance to tetracycline

As mentioned above, 31% of the genes de-repressed during iron starvation in ***Δ*** *hapX* encode mitochondrial-localized proteins and 66% (27 genes) of those are up-regulated in ***Δ*** *sreA* during iron sufficiency (Table 1A and Table S1 in Supporting Information S1), which indicates a major impact of iron de-regulation on mitochondrial metabolism. Live cell imaging by laser scanning confocal microscopy of the mitotracker-stained mitochondrial network revealed no differences between *wt*, ***Δ*** *hapX*, and ***Δ*** *sreA* neither during iron-replete nor iron-depleted conditions (data not shown). Next we analyzed the mtDNA content of wt, ***Δ*** *hapX* and ***Δ*** *sreA* by qPCR normalized against the content of nuclear DNA (Table S7 in Supporting Information S1). Concomitant with derepression of genes encoding mitochondrial proteins, HapX deficiency increased the mtDNA content during iron starvation 1.9-fold but had no effect during iron sufficiency. *Vice versa*, SreA-deficiency increased the mtDNA content during iron sufficiency 2.3-fold but had no effect during iron starvation. Little is known about the molecular mechanisms coordinating replication of nuclear DNA and mtDNA in *Aspergilli*. Inactivation of SreA and HapX, respectively, may disturb this coordination by deregulation of either the general mitochondrial metabolism (proteins and/or metabolites) and/or of a specific regulator. Notably, ***Δ*** *hapX* and ***Δ*** *sreA* display decreased growth rates under the conditions, in which they have increased mtDNA contents (see above and [22]). Formally, it is therefore also possible that toxic effects caused by deficiency in SreA and HapX slow down nuclear DNA replication, whereby the deregulation of nuclear-encoded mitochondrial proteins disturbs the coordination with mitochondrial replication. HapX-deficiency also decreased resistance to tetracycline, an inhibitor of bacterial and mitochondrial protein synthesis [31], during iron-depleted but not iron-replete conditions (Fig. 2A), which underlines that HapX-deficiency affects mitochondrial metabolism.

### HapX inactivation increases zinc sensitivity

We have previously shown that there is a close connection between zinc and iron metabolism [32]. In order to avoid zinc excess and zinc toxicity, iron starvation down-regulates expression of genes encoding plasma membrane zinc transporters such as *zrfB* (Afu2g03860) and the respective transcription activator *zafA* (Afu1g10080) and concomitantly up-regulates the vacuolar zinc/cadmium transporters *zrcA* (Afu7g06570) and *cotA* (Afu2g14570). The expression profiling indicated increased expression of *zrfB* and *zafA* (Fig. S1) and decreased expression of *zrcA* and *cotA* (Fig. S2) in ***Δ*** *hapX* during iron starvation suggesting increased zinc uptake and decreased vacuolar zinc storage. In agreement, ***Δ*** *hapX* displayed increased sensitivity to zinc (Fig 2E), which indicates a role of HapX in coordination of iron and zinc homeostasis.

### HapX is crucial for virulence in a murine model of invasive aspergillosis

To determine whether HapX-mediated regulation is relevant for growth of *A. fumigatus* in the environment of the host, we compared the virulence of the ***Δ*** *hapX* strain with that of the complemented ***Δ*** *hapX^c^* strain and the *wt* strain in two different mouse models of pulmonary invasive aspergillosis: (i) a leucopenic mouse model using immunosuppression with both cortisone acetate and cyclophosphamide [33], [34], and (ii) a non-leucopenic model with immunosuppression by cortisone acetate [35], [36]. In the leucopenic host, a cellular immune response is virtually absent and development of invasive aspergillosis is characterized by extensive invasive growth of the fungus [37]. Thus, this model allows assessing whether fungal factors are required for survival and growth on lung tissue in general. In contrast, the cortisone acetate model allows recruitment of neutrophils and monocytes, which, despite partially impaired phagocytosis, attack fungal cells and prevent rapid fungal dissemination [38]. Mice were infected with 1×10^5^ conidia in the leucopenic mouse model and 1×10^6^ conidia in the cortisone acetate mouse model to account for the decreased killing rate; survival was monitored over a period of 14 days, followed by histological analyses of the lungs.

**Figure 5:**
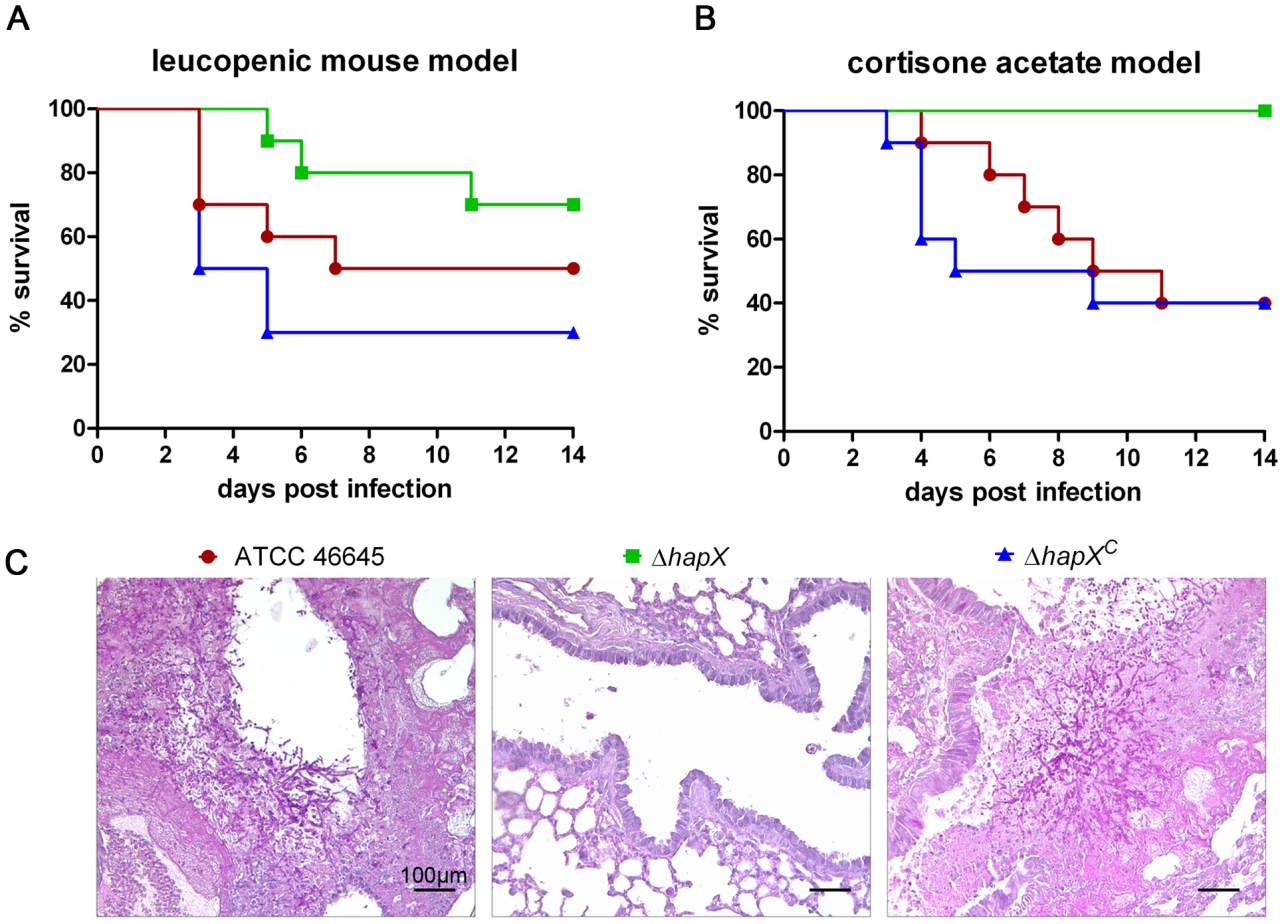
HapX-deficiency results in attenuation of *A. fumigatus* virulence. Survival of leucopenic mice (A) and mice immunosuppressed with cortisone acetate (B) after infection with Δ*hapX*, the complemented strain Δ*hapX^C^*, or *wt*. (C) Histopathology using PAS staining (hyphae stain pink) of leucopenic mice infected with Δ*hapX*, Δ*hapX^C^*, or *wt*.

As shown in the survival curves in Fig. 5A both *wt* and ***Δ*** *hapX^c^* caused high mortality rates in the leucopenic mouse model, which were statistically not significantly different (p = 0.29) by Kaplan-Meyer estimation and log rank tests. The ***Δ*** *hapX* mutant displayed attenuation in virulence, which was however statistically significant only compared to ***Δ*** *hapX^c^* (p = 0.033) but not compared to *wt* (p = 0.28). At necropsy, the reduced virulence of ***Δ*** *hapX* was reflected in the incidence of macroscopic lung alterations in comparison to both ***Δ*** *hapX^c^* and *wt* (Fig. 5C): eight of ten mice infected with ***Δ*** *hapX^c^*, seven of ten mice infected with *wt*, but only one of ten mice infected with ***Δ*** *hapX* displayed lung alterations. The presence of invasive mycelia could be confirmed in the majority of mice infected with ***Δ*** *hapX^c^* and *wt* but no mycelium could be found in any mouse infected with ***Δ*** *hapX* (Fig. 5C).

In the cortison acetate mouse model, no statistically significant difference in survival were detected between mice infected with either *wt* or ***Δ*** *hapX^c^* (p = 0.67). In contrast, ***Δ*** *hapX* was completely attenuated compared to both *wt* and ***Δ*** *hapX^c^* (p = 0.004). Consistently, the lungs of all 10 mice infected with ***Δ*** *hapX* were unaltered whereas the lungs of six of ten mice infected with ***Δ*** *hapX^c^* showed clear symptoms of inflammation (data not shown).

The expression of *hapX* is repressed by iron (see above), and, consistently, deleterious effects of *hapX*-inactivation are limited to iron-starved conditions (see above). Therefore, the attenuated virulence of ***Δ*** *hapX* is in agreement with *A. fumigatus* facing iron-limited conditions in the host and the requirement of HapX for virulence. This is also in accordance with the importance of the iron-repressed siderophore system and the dispensability of the iron-induced iron regulator SreA for pathogenicity [5], [10], [22]. Notably, supplementation with iron-free TAFC or FsC to a final concentration of 10 mM did neither cure the growth defect nor inhibit the PpIX accumulation of ***Δ*** *hapX* during iron starvation in liquid flask cultures (data not shown) indicating that the reduced TAFC production does not account for the full extent of the ***Δ*** *hapX* phenotype. Together with the fact that HapX-deficiency causes decreased production of TAFC but not FsC (see above) and the previous finding that the *A. fumigatus* ***Δ*** *sidG* mutant strain, which produces FsC but not TAFC, displays unaltered virulence in a mouse model for pulmonary aspergillosis [10], these data suggest that the reduced virulence of ***Δ*** *hapX* is not caused, at least not solely, by the decreased TAFC production. Therefore, the attenuated virulence of ***Δ*** *hapX* might be caused by the general deregulation of gene expression (i.e. the missing metabolic adaption to iron starvation), the accumulation of toxic metabolites such as PpIX, and/or the down-regulation of possible virulence determinants such as AspF1 (see above).

The ***Δ*** *hapX* mutant appeared to be slightly more virulent in the leucopenic mouse model compared to the cortisone-acetate model (Fig. 5A and B). As HapX-deficiency results in sensitivity to iron starvation, these data indicate that the attack of neutrophils and monocytes, which is absent in the leucopenic model, increases extracellular iron starvation or imposes iron starvation by internalization. In this respect it is interesting to note that the siderophore system was recently shown to play a crucial role in intracellular growth and survival in murine alveolar macrophages demonstrating that *A. fumigatus* faces iron starvation after phagocytosis [39]. In agreement, the siderophore system was shown to be essential to alter immune effector pathways and iron homeostasis of murine macrophages [40].

Apart from *A. fumigatus* HapX, only one fungal iron regulator has been shown to be required for virulence so far: *C. neoformans* Cir1, the ortholog of *A. fumigatus* SreA [41]. Similar to SreA-deficiency in *A. fumigatus*, Cir1-deficiency impairs growth during iron-replete but not depleted conditions, which does not implicate a crucial role in virulence at first sight. But in contrast to *A. fumigatus* SreA, which is not required for virulence [22], *C. neoformans* Cir1 functions also as an activator for growth at 37°C (host temperature) and capsule formation, which are both important virulence traits.

### Conclusions

This study demonstrates that the metabolic reprogramming required for adaption to iron starvation depends on HapX and that this adaption is essential for virulence of *A. fumigatus*. The identification of numerous HapX-affected genes with yet uncharacterized link to iron or starvation will aid in the further characterization of the metabolic pathways required for adaption to iron starvation and consequently virulence traits of *A. fumigatus*. This study appears to be exemplary for the iron metabolism and virulence of most fungal species as HapX is widely conserved with exception of species closely related to *S. cerevisiae*.

## Methods

### Fungal strains, growth conditions

Fungal strains used were *A. fumigatus* wild-type ATCC46645 (American Type Culture Collection ), ***Δ*** *sreA* (*ATCC46645*, ***Δ*** *sreA::hph*), ***Δ*** *sidA* (*ATCC46645*, ***Δ*** *sidA::hph*), ***Δ*** *hapX* (*ATCC46645*, ***Δ*** *hapX::hph*), and ***Δ*** *hapX^C^* (***Δ*** *hapX*, *(p)::hapX*). ***Δ*** *sreA* and ***Δ*** *sidA* were described previously [5], [22]; generation of ***Δ*** *hapX* and ***Δ*** *hapX^C^* is described below. Generally, *A. fumigatus* strains were grown at 37°C in *Aspergillus* minimal medium according to Pontecorvo et al. [42] containing 1% glucose as the carbon source and 20 mM glutamine as the nitrogen source. Iron-replete media contained 30 mM FeSO_4_. For iron depleted conditions, iron was omitted. The BPS and tetracycline concentrations used were 200 µM and 2 mg ml^−1^ respectively. For growth assays, 10^4^ and 10^8^ conidia were used for point-inoculation on plates or inoculation of 100 ml liquid media, respectivly.

### Northern analysis and DNA manipulations

RNA was isolated using TRI Reagent (Sigma). For Northern analysis, 10 µg of total RNA was analyzed as described previously [43]. Hybridization probes and Primers used are listed in Table S7 in Supporting Information S1. For extraction of genomic DNA, mycelia were homogenized and DNA was isolated according to Sambrook et al. [44]. For general DNA propagations *Escherichia coli* DH5α strain was used as a host.

### Deletion of *hapX* and complementation of the *ΔhapX* strain

For generating the *ΔhapX* mutant strain, the bipartite marker technique was used [45]. Briefly, *A. fumigatus* was co-transformed with two DNA fragments, each containing overlapping but incomplete fragments of the pyrithiamine resistance-conferring *ptrA* gene fused to 1.2-kb *hapX* 5′- and 3′-flanking sequences, respectively. The *hapX* 5′-flanking region (1207bp) was PCR-amplified from genomic DNA using primers ohapX-1 and ohapX-4. For amplification of the 3′flanking region (1156bp) primers ohapX-2 and ohapX-3 were employed. Subsequent to gel-purification, these fragments were digested with *Sac*I (5′flanking region) and *Xho*I (3′flanking region), respectively. The *ptrA* selection marker was released from plasmid pSK275 by digestion with *Sma*I and *Xho*I, respectively, and ligated with the 5′- and 3′-flanking region, respectively. The transformation construct A (2558bp, fusion of the *hapX* 5′-flanking region and the *prtA* split marker) was amplified from the ligation product using primers ohapX-5 and optrA-2. For amplification of the transformation construct B (2166bp, fusion of the *hapX* 3′-flanking region and the supplementary *prtA* split marker) primers ohapX-6 and optrA-1 were employed. For transformation of *A. fumigatus* ATCC46645 both constructs A and B were simultaneously used. This strategy deleted the sequence −228 to 1383 bp relative to the translation start site in *hapX*.

For the reconstitution of the *ΔhapX* strain with a functional *hapX* copy, a 3615bp PCR fragment generated with primers ohapX-5 and ohapX-6 was subcloned into pGEM-T (Promega) according to the supplier's manual, resulting in pHapX. A 2410bp *Sph*I fragment from pAN7-1 containing the hygromycine B resistance-conferring *hph* gene was inserted into the *Sph*I site of pHapX resulting in pHapXhph. The resulting 9.0-kb plasmid pHapXhph was linearized with *Eco*RV and used to transform *A. fumigatus ΔhapX*.

Transformation of *A. fumigatus* was carried out as described previously [10]. For selection of transformants 0.1 µg ml^−1^ pyrithiamine (Sigma) or 0.2 mg ml^−1^ hygromycin B (Calbiochem) was used. Screening of transformants was performed by PCR and confirmed by Southern blot analysis. The hybridization probes for Southern blot analysis of *ΔhapX* and ***Δ*** *hapX^c^* strains were generated by PCR using the primers ohapX-5 and ohapX-4 (Table S8 in Supporting Information S1).

### Analysis of siderophores, PpIX, and free amino acids

Analysis of siderophore, PpIX and free amino acids was carried out by reversed phase HPLC as described previously [20], [43], [46]. To quantify extracellular or intracellular siderophores, culture supernatants or cellular extracts were saturated with FeSO_4_ and siderophores were extracted with 0.2 volumes of phenol. The phenol phase was separated and subsequent to addition of 5 volumes of diethylether and 1 volume of water, the siderophore concentration of the aqueous phase was measured photometrically using a molar extinction factor of 2996/440nm (M^−1^cm^−1^).

### Determination of mtDNA content

*A. fumigatus* total DNA was isolated with the QIAamp kit (Qiagen). MtDNA compared to nuclear DNA content was determined by quantitative real-time PCR (qPCR) with CYBR green I (ABI; ABI 2400 Applied Biosystems, USA) by PCR amplification of a fragment of the mitochondrial apocytochrome B gene (BAA34151, 73.t00020) using primers oAfcytB-1 and oAfcytB-2, and a fragment of the nuclear *mirD* gene (Afu3G03440) gene, using primers oAfmirD-1 and oAfmirD-2. The PCR reactions cycle used (Applied Biosystems standard conditions) was 40 cycles at 95°C 15″, 60°C 1′. PCR assays were performed in biological triplicates and technical duplicates for each DNA sample. The expression of mtDNA copy number relative to nuclear DNA was determined using the 2^−ΔCT^ method.

### Transcriptional profiling

The *A. fumigatus* Af293 DNA amplicon microarray containing 9,516 genes [47] was used in this study. To profile the genome-wide expression responses to the shift from iron depleted to iron-replete conditions and to identify the genes influenced by HapX, we conducted microarray analysis with the *wt* and ***Δ*** *hapX* strains grown for 16 h at 37°C in iron-depleted (−Fe) medium (0 h time point). Subsequently iron was added to a final concentration of 30 mM and growth was continued for 1 hour (sFe). Labelling reactions with RNA, and hybridization were conducted as described in the PFGRC standard operating procedures (PFGRC SOP's) found at http://pfgrc.tigr.org/protocols/protocols.shtml. The sample from 0 h served as reference in all hybridizations with the 1 h iron shift samples in order to identify genes exhibiting altered transcription after the shift from iron depleted to iron replete conditions in *ΔhapX* compared to *wt*. Additionally, the 16 h iron starvation cultures of *ΔhapX* and wt were directly compared with the wt serving as reference. (Fig. S1 and S2)

Hybridized slides were scanned using the Axon GenePix 4000B microarray scanner and the TIFF images generated were analyzed using the TM4 suite of microarray analysis tools (http://www.tm4.org). Spotfinder was used to obtain relative transcript levels. Data from Spotfinder were stored in MAD, a relational database designed to effectively capture and store microarray data. Data normalization was accomplished using a local regression technique LOWESS (LOcally WEighted Scatterplot Smoothing) for hybridizations using the TM4 MIDAS tool. The resulting data was averaged from triplicate gene spots on each array and from duplicate flip-dye arrays for each experiment, taking a total of 6 intensity data points for each gene. Differentially expressed genes at the 95% confidence level were determined using intensity-dependent Z-scores (with *Z* = 1.96) as implemented in MIDAS and the union of all genes identified at each time point from the wild-type were considered significant in this experiment. Microarray data are deposited in the GEO database (http://www.ncbi.nlm.nih.gov/geo/query/acc.cgi?token=jhqbdcwiigqyypa&acc=GSE22052).

### Virulence assay

Virulence assays in two murine models for pulmonary aspergillosis were performed as described previously [33], [34]. Infections were performed with two groups of five mice for each tested strain. A control group remained uninfected (inhalation of PBS) to monitor the influence of the immunosuppressive regime. Survival data were plotted as Kaplan-Meyer curves and were analyzed statistically by a log rank test using Graph Pad Prism version 5.00 for Windows (GraphPad Software, San Diego, CA). Lungs from euthanized animals were removed, fixed in formalin and paraffin-embedded for histopathologic analyses according to standard protocols. Sections were stained with Periodic acid-Schiff (PAS) according to standard protocols and analyzed by bright field microscopy using a Zeiss AxioImager.M1 microscope equipped with a SPOT Flex Shifting Pixel Color Mosaic camera (Diagnostic Instruments, Inc., Sterling Heights, USA).

### Ethics statement

Mice were cared for in accordance with the principles outlined by the European Convention for the Protection of Vertebrate Animals Used for Experimental and Other Scientific Purposes (European Treaty Series, no. 123; http://conventions.coe.int/Treaty/en/Treaties/Html/123.htm). All animal experiments were in compliance with the German animal protection law and were approved (permit no. 03-001/08) by the responsible Federal State authority (Thüringer Landesamt für Lebensmittelsicherheit und Verbraucherschutz) and ethics committee (beratende Komission nach § 15 Abs. 1 Tierschutzgesetz).

## Supporting Information

Figure S1 Biosynthesis of both TAFC and FC starts with N5-hydroxylation of ornithine. Subsequently, the hydroxamate group is formed by the transfer of an acyl group from acyl-coenzyme A (CoA) derivatives to N5-hydroxyornithine. Here the pathways for biosynthesis of TAFC and FC split due to the choice of the acyl group with acetyl for FC and anhydromevalonyl for TAFC. Assembly of the cyclic siderophores fusarinine C and FC is catalysed by different non-ribosomal peptide synthetases (NRPS). TAFC and hydroxyferricrocin are formed by N2-acetylation of fusarinine C and hydroxylation of FC respectively. With exception of the acetyl transferase required for FC biosynthesis all A. fumigatus genes encoding respective enzyme activities have been identified and are indicated (Haas et al., 2008; Schrettl et al., 2004; Schrettl et al., 2007).
(0.55 MB TIF)

Figure S2 *A. fumigatus* wt was grown for 24 h at 37°C in liquid flask cultures and 10 ml of culture supernatant was analyzed by reversed phase HPLC.
(0.10 MB TIF)

Supporting Information S1 Tables S1 to S8.
(6.06 MB DOC)
