# Supplementary figures and images for: HapX-Mediated Adaption to Iron Starvation Is Crucial for Virulence of Aspergillus fumigatus
Source: PLoS Pathog. 2010 Sep 30;6(9):e1001124. doi: 10.1371/journal.ppat.1001124 (PMC2947994; doi:10.1371/journal.ppat.1001124)

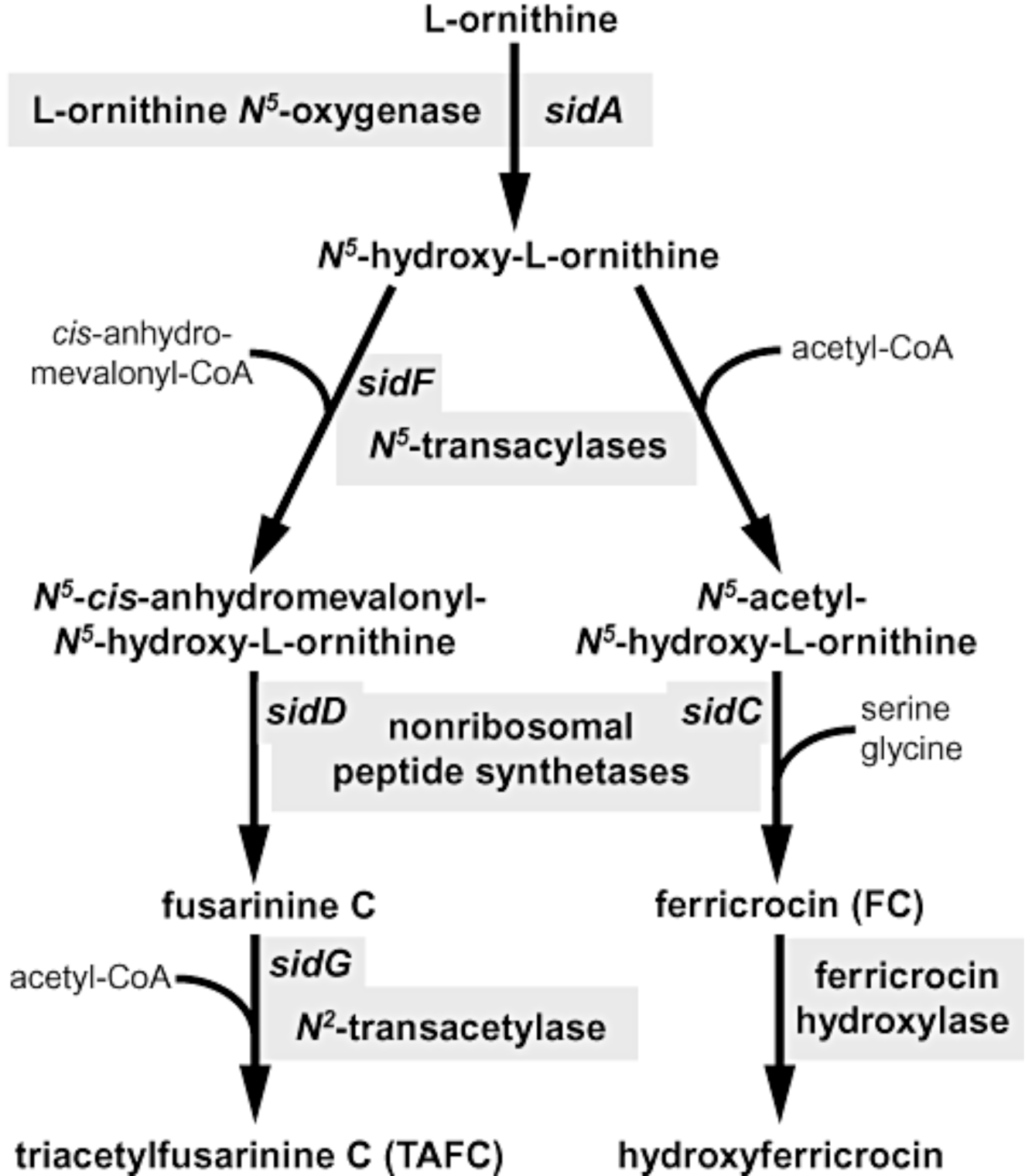

Supplement: Figure S1 — Biosynthesis of both TAFC and FC starts with N5-hydroxylation of ornithine. Subsequently, the hydroxamate group is formed by the transfer of an acyl group from acyl-coenzyme A (CoA) derivatives to N5-hydroxyornithine. Here the pathways for biosynthesis of TAFC and FC split due to the choice of the acyl group with acetyl for FC and anhydromevalonyl for TAFC. Assembly of the cyclic siderophores fusarinine C and FC is catalysed by different non-ribosomal peptide synthetases (NRPS). TAFC and hydroxyferricrocin are formed by N2-acetylation of fusarinine C and hydroxylation of FC respectively. With exception of the acetyl transferase required for FC biosynthesis all A. fumigatus genes encoding respective enzyme activities have been identified and are indicated (Haas et al., 2008; Schrettl et al., 2004; Schrettl et al., 2007). (0.55 MB TIF) [file ppat.1001124.s001.tif]

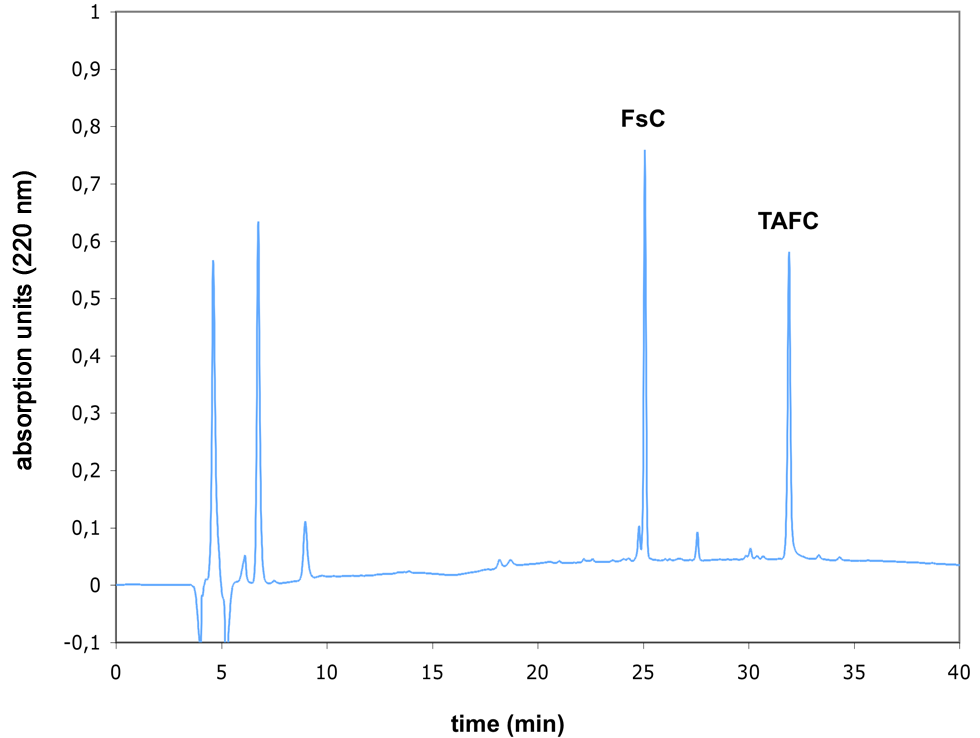

Supplement: Figure S2 — A. fumigatus wt was grown for 24 h at 37°C in liquid flask cultures and 10 ml of culture supernatant was analyzed by reversed phase HPLC. (0.10 MB TIF) [file ppat.1001124.s002.tif]
