# Supplementary material for: HapX-Mediated Adaption to Iron Starvation Is Crucial for Virulence of Aspergillus fumigatus
Source: PLoS Pathog. 2010 Sep 30;6(9):e1001124. doi: 10.1371/journal.ppat.1001124 (PMC2947994; doi:10.1371/journal.ppat.1001124)
Supplement: Supporting Information S1 — Tables S1 to S8. (6.06 MB DOC) [file ppat.1001124.s003.doc]

**Table S1 in Supporting Information S1. Genes derepressed in *ΔhapX*.**

Listed genes fulfilled three criteria in microarray-based transcriptional profiling: (i) > 1.5-fold up-regulation in a 1h-shift from iron starvation to iron sufficiency in *wt* (column ***w*/sFe**), (ii) > 1.5-fold decreased up-regulation in a 1h-shift from iron starvation to iron sufficiency in ***∆****hapX* compared to *wt* (column***h-w*/sFe**), and (iii) >1.5-fold up-regulation during steady-state iron starved growth in ***∆****hapX* compared to *wt* (column ***hw*/-Fe**)*.* Microarray data displayed: ***w*/sFe**, 1h-iron shift versus iron starvation of *wt*; ***h*/sFe**, ***∆****hapX* 1h-iron shift versus iron starvation of ***∆****hapX;* ***w-h*/sFe**, comparison of ***w*/sFe** versus ***h*/sFe**; ***hw*/-Fe**, ***∆****hapX* versus *wt* during iron starvation. Values are given in Log_2_ scale and represent the median of two biological replicates with the 1.5-fold difference occurring in both replicates. Categorized genes are listed in the order of their chromosomal localization. Changes > and < 1.5-fold are marked in pinc and green, respectively. Genes previously identified to be indirectly up-regulated in ***∆****sreA* (see text) are marked in pink in the column SreAi [1]. Mitochondrial genes are marked in red in the column **M**. Membrane proteins are marked in yellow in the colums **T**. Genes previously identified to be negatively affected by Php4 in *S. pombe* [2] are marked in blue. Genes previously identified to be negatively affected by Cth1 or Cth2 in *S. cerevisae* [3] are marked in orange. Accession numbers of genes organized in gene clusters are boxed and listed collectively in Table S3.

**Table S2 in Supporting Information S1. Genes positively affected by HapX.**

Listed genes fulfilled three criteria in microarray-based transcriptional profiling: (i) > 1.5-fold down-regulation in a 1h-shift from iron starvation to iron sufficiency in *wt* (column ***w*/sFe**), (ii) > 1.5-fold decreased down-regulation in a 1h-shift from iron starvation to iron sufficiency in ***∆****hapX* compared to *wt* (column***w-h*/sFe**), (iii) >1.5-fold down-regulation during steady-state iron starved growth in ***∆****hapX* compared to *wt* (column ***hw*/-Fe**)*.* Microarray data displayed: ***w*/sFe**, 1h-iron shift versus iron starvation of *wt*; ***h*/sFe**, ***∆****hapX* 1h-iron shift versus iron starvation of ***∆****hapX;* ***w-h*/sFe**, comparison of ***h*/sFe** versus ***w*/sFe**; ***hw*/sFe**, ***∆****hapX* versus *wt* during iron starvation. Values are given in Log_2_ scale and represented the median of two biological replicates with the 1.5-fold difference occurring in both replicates. Categorized genes are listed in the order of their chromosomal localization. Changes > and < 1.5-fold are marked in pink and green, respectively. Genes previously identified to be likely targets of SreA [1] are marked in pink in the column **SreA**. Mitochondrial Genes are marked in red in the column **M**. membrane proteins are marked in yellow in the column **T**. Accession numbers of genes organized in gene clusters are boxed and listed collectively in Table S4.

**Table S3 in Supporting Information S1. Gene clusters negatively affected by HapX**

| Afu2g10490 | hypothetical protein |
| --- | --- |
| Afu2g10500 | ribosomal protein S9 |
|  |  |
| Afu2g13710 | NADH-ubiquinone oxidoreductase 49 kDa subunit |
| Afu2g13720 | RNA-polymerase I |
|  |  |
| Afu3g03610 | glycosyl hydrolase family 12 protein |
| Afu3g03620 | glycosyl transferase |
|  |  |
| Afu4g11270 | hypothetical protein |
| Afu4g11280 | GPI mannosyltransferase (GPI18, PIG-V) |
|  |  |
| Afu4g11390 | ubiquinol-cytochrome c reductase complex 17 kd protein |
| Afu4g11400 | MFS heme transporter (FLVCR) |
|  |  |
| Afu5g07480 | hypothetical protein |
| Afu5g07490 | steroid monooxygenase |
|  |  |
| Afu6g09190 | metallopeptidase family M24 |
| Afu6g09200 | COG3467 (flavin-binding protein family) |
|  |  |
| Afu6g12920 | C2H2 finger domain protein (Bud20) |
| Afu6g12930 | aconitase |

**Table S4 in Supporting Information S1.Gene clusters positively affected by HapX.**

| Afu1g03330 | hypothetical protein (pseudogene?) |
| --- | --- |
| Afu1g03352 | alpha-1,3-glucanase |
| Afu1g03360 | hypothetical protein |
|  |  |
| Afu1g17180 | **pyridine nucleotide-disulphide oxidoreductase** |
| Afu1g17190 | **long-chain-fatty-acid-CoA ligase SidI** |
| Afu1g17200 | **ferricrocin NRPS SidC** |
|  |  |
| Afu2g05720 | **conserved hypothetical protein (DUF1349)** |
| Afu2g05730 | **siderochrome-iron transporter (MirC)** |
|  |  |
| Afu2g11340 | ML domain protein (lipid metabolism) |
| Afu2g11350 | peroxisomal 3-ketoacyl-coA thiolase (Kat1) |
|  |  |
| Afu2g13460 | alpha-amylase, putative |
| Afu2g13470 | pyrimidine 5'-nucleotidase |
|  |  |
| Afu2g15380 | hypothetical protein |
| Afu2g15390 | sporulation regulator FluG |
|  |  |
| Afu3g02260 | hypothetical protein |
| Afu3g02250 | hydroxysteroid dehydrogenase/isomerase (Gre2) |
|  |  |
| Afu3g03650 | **acetyltransferase SidG (TAFC biosynthesis)** |
| Afu3g03670 | **ABC multidrug transporter** |
|  |  |
| Afu4g08370 | conserved hypothetical protein |
| Afu4g08380 | hypothetical protein |
|  |  |
| Afu5g00700 | hypothetical protein |
| Afu5g00710 | GABA permease, putative |
| Afu5g00720 | GNAT family, acetyltransferase |
|  |  |
| Afu5g02320 | hypothetical protein |
| Afu5g02330 | major allergen Asp F1 |

Clusters containing siderophore biosynthetic genes are in bold.

**Table S5 in Supporting Information S1. Free amino acid pools of *A. fumigatus wt, ∆hapX, ∆sreA* and *∆sidA* under iron replete and depleted conditions.**

| aa | ***wt***  **+Fe** | ***wt***  **-Fe** | ***wt*^1^**  **-/+Fe** | ***∆hapX*^2^**  **+Fe** | ***∆hapX*^2^**  **-Fe** | ***∆hapX*^2^**  **-/+Fe** | ***∆sreA*^2^**  **+Fe** | ***∆sreA*^2^**  **-Fe** | ***∆sreA*^2^**  **-/+Fe** | ***∆sidA*^2^**  **+Fe** | ***∆sidA*^2^**  **-Fe** | ***∆sidA*^2^**  **-/+Fe** |
| --- | --- | --- | --- | --- | --- | --- | --- | --- | --- | --- | --- | --- |
| Ala | 34.15±0.93 | 8.63±1.29 | 0.25 | 36.59±2.69 | 3.55±0.23 | 0.10 | 33.52±3.33 | 9.67±2.00 | 0.29 | 31.45±5.30 | 6.74 ±0.65 | 0.21 |
| Arg | 1.28±0.11 | 13.20±1.75 | 10.31 | 1.59±0.06 | 20.96±2.59 | 13.21 | 1.08±0.09 | 14.89±0.12 | 13.76 | 1.56±0.18 | 14.94±2.16 | 9.56 |
| Asn | 0.82±0.04 | 2.46±0.29 | 3.01 | 0.86±0.05 | 3.53±0.20 | 4.10 | 1.07±0.09 | 2.53±0.24 | 2.37 | 0.73±0.04 | 2.07±0.44 | 2.83 |
| Asp | 3.61±0.40 | 3.47±0.33 | 0.96 | 4.12±0.42 | 4.01±0.32 | 0.97 | 4.69±1.35 | 3.63±1.05 | 0.77 | 3.24±0.92 | 2.26±0.96 | 0.70 |
| Gln | 7.03±0.25 | 37.01±2.75 | 5.26 | 6.32±0.72 | 21.29±2.69 | 3.37 | 5.10±1.14 | 33.57±3.13 | 6.58 | 7.36±0.95 | 31.09±9.22 | 4.22 |
| Glu | 42.14±1.46 | 18.29±0.54 | 0.43 | 36.62±3.58 | 17.03±0.71 | 0.47 | 41.47±2.65 | 18.62±2.29 | 0.45 | 44.18±6.51 | 14.36±4.35 | 0.33 |
| Gly | 1.53±0.10 | 1.03±0.02 | 0.67 | 1.81±0.10 | 7.63±1.25 | 4.22 | 1.52±0.04 | 1.13±0.07 | 0.74 | 1.55±0.26 | 0.51±0.15 | 0.33 |
| His | 0.18±0.06 | 1.69±0.10 | 9.17 | 0.32±0.08 | 3.32±0.22 | 10.50 | 0.24±0.03 | 1.70±0.17 | 7.21 | 0.20±0.07 | 1.44±0.40 | 7.14 |
| Ile | 0.34±0.01 | 0.33±0.07 | 1.00 | 0.43±0.01 | 0.40±0.06 | 0.94 | 0.41±0.03 | 0.34±0.03 | 0.83 | 0.29±0.06 | 0.21±0.06 | 0.74 |
| Leu | 0.38±0.05 | 0.50±0.06 | 1.33 | 0.53±0.02 | 0.80±0.12 | 1.51 | 0.50±0.05 | 0.45±0.06 | 0.91 | 0.39±0.02 | 0.32±0.06 | 0.82 |
| Lys | 1.55±0.20 | 3.45±0.32 | 2.23 | 1.86±0.18 | 10.82±0.91 | 5.82 | 1.49±0.20 | 3.60±0.18 | 2.42 | 1.56±0.30 | 2.41±0.56 | 1.54 |
| Met | 0.06±0.01 | 0.09±0.02 | 1.68 | 0.07±0.02 | 0.07±0.01 | 1.01 | 0.06±0.02 | 0.10±0.04 | 1.62 | 0.06 ±0.00 | 0.06±0.03 | 1.04 |
| Orn | 0.76±0.01 | 5.24±0.49 | 6.91 | 1.43±0.12 | 0.42±0.13 | 0.30 | 0.29±0.02 | 5.16±0.20 | 17.77 | 1.42±0.21 | 20.08±2.30 | 14.15 |
| Phe | 0.11±0.03 | 0.18±0.03 | 1.63 | 0.15±0.01 | 0.30±0.04 | 1.95 | 0.13 ±0.01 | 0.16±0.01 | 1.24 | 0.11±0.02 | 0.15±0.04 | 1.40 |
| Ser | 1.92±0.04 | 1.65±0.09 | 0.86 | 2.11±0.05 | 2.35±0.15 | 1.11 | 2.12±0.10 | 1.61±0.11 | 0.76 | 2.09±0.06 | 1.17±0.34 | 0.56 |
| Thr | 1.44±0.06 | 1.51±0.09 | 1.05 | 1.77±0.34 | 1.80±0.28 | 1.01 | 2.10±0.31 | 1.65±0.21 | 0.78 | 1.34±0.30 | 1.22±0.35 | 0.91 |
| Trp | 0.03±0.02 | 0.04±0.02 | 1.55 | 0.04±0.02 | 0.11±0.03 | 3.14 | 0.03±0.01 | 0.04±0.01 | 1.34 | 0.02±0.02 | 0.04±0.02 | 1.98 |
| Tyr | 0.25±0.07 | 0.37±0.04 | 1.47 | 0.34±0.04 | 0.53±0.08 | 1.56 | 0.34±0.03 | 0.35±0.04 | 1.04 | 0.25±0.05 | 0.45±0.08 | 1.77 |
| Val | 2.44±0.03 | 0.85±0.22 | 0.35 | 3.05±0.18 | 1.08±0.18 | 0.35 | 3.84±0.32 | 0.80±0.14 | 0.21 | 2.20±0.36 | 0.48±0.10 | 0.22 |

Individual amino acid pools are given in % of the total free amino acids.

***Table S6 in Supporting Information S1. Comparison of the –Fe/+Fe ratios of free amino acid pool of A. fumigatus and*** S. cerevisiae

| **aa** | **-Fe/+Fe** | |
| --- | --- | --- |
|  | ***S. cerevisiae*** | ***A. fumigatus*** |
| Gly | 2.4 | 0.7 |
| Thr | 1.6 | 1.1 |
| Ser | 0.9 | 0.9 |
| Ala | 1.7 | 0.3 |
| Asp | 1 | 1.0 |
| Asn | 1.7 | 3.0 |
| Glu | 0.7 | 0.4 |
| Gln | 1 | 5.3 |
| His | 1.3 | 9.2 |
| Lys | 0.8 | 2.2 |
| Phe | 1.3 | 1.6 |
| Tyr | 1.3 | 1.5 |
| Trp | 1.7 | 1.6 |
| Ile | 1.6 | 1.0 |
| Leu | 1.8 | 1.3 |
| Val | 1.7 | 0.4 |
| Cys | 1.3 | nd |
| Met | 1.1 | 1.7 |
| Arg | 1.1 | 10.3 |
| Pro | 0.9 | nd |
| Orn | nd | 6.9 |

Amino acids up-regulated > 1.5- and > 3-fold during iron starvation compared to sufficiency (-Fe/+Fe) are marked in and , respectively; amino acids down-regulated > 1.5- and > 3-fold are marked in and , respectively. *nd* is not determined.

**Table S7 in Supporting Information S1. The mtDNA content is increased by HapX-deficiency during iron starvation (-Fe) and SreA-deficiency during iron sufficiency (+Fe).**

|  | **-Fe** | **+Fe** |
| --- | --- | --- |
| **wt** | 1.0±0.04 | 0.90±0.04 |
| ***∆hapX*** | 1.88±0.25 | 1.10±0.03 |
| ***∆sreA*** | 1.13±0.10 | 2.28±0.22 |

mtDNA content was measured by real-time PCR and normalized with nuclear DNA. The results are expressed as a ratio relative to the *wt* during iron starvation.

**Table S8 in Supporting Information S1. Primers used in this study**

| **Primers** | **sequence 5´-3** |
| --- | --- |
| oAfhapX-1 | AGC GAC TAT AGC CGG ATG |
| oAfhapX-2 | CCT TGG GTC TTG AAG CTT GCG |
| oAfhapX-3 | TCA TCT AGA CTG CCC AAG CTT CAT ACC |
| oAfhapX-4 | ATC AGA GCT GGA GAG GCA |
| oAfhapX-5 | TGG AGT TCC GAT TGG TGC |
| oAfhapX-6 | ATC CCG CTT CTT TCA CCC |
| oAfcytB-1 | TGTATTCTTCATGCCTAACGC |
| oAfcytB-2 | ATCATTCCGGAACAATAGCA |
| oAfmirD-1 | AGAGCAAGATGTTGATTGCG |
| oAfmirD-1 | TCCCGAATAACAAACATCCA |
|  |  |
|  |  |

1. Schrettl M, Kim HS, Eisendle M, Kragl C, Nierman WC, et al. (2008) SreA-mediated iron regulation in Aspergillus fumigatus. Mol Microbiol 70: 27-43.

2. Mercier A, Watt S, Bahler J, Labbe S (2008) Key function for the CCAAT-binding factor Php4 to regulate gene expression in response to iron deficiency in fission yeast. Eukaryot Cell 7: 493-508.

3. Puig S, Vergara SV, Thiele DJ (2008) Cooperation of two mRNA-binding proteins drives metabolic adaptation to iron deficiency. Cell Metab 7: 555-564.
